# Endangered Schizothoracin Fish in the Tarim River Basin Are Threatened by Introgressive Hybridization

**DOI:** 10.3390/biology11070981

**Published:** 2022-06-28

**Authors:** Lei Cheng, Dan Song, Xiaoli Yu, Xue Du, Tangbin Huo

**Affiliations:** Key Laboratory of Freshwater Aquatic Biotechnology and Breeding, Ministry of Agriculture and Rural Affairs, Heilongjiang River Fisheries Research Institute, Chinese Academy of Fishery Sciences, Harbin 150070, China; chenglei@hrfri.ac.cn (L.C.); songdan@hrfri.ac.cn (D.S.); yuxiaoli0311@163.com (X.Y.)

**Keywords:** *Aspiorhynchus laticeps*, *Schizothorax biddulphi*, *Schizothorax esocinus*, mitochondrial DNA (mtDNA), internal transcribed spacer 2 (ITS2), hybridization, introgression

## Abstract

**Simple Summary:**

Interspecific hybridization and introgression may threaten the survival of endangered species. Big-head Schizothoracin (*Aspiorhynchus laticeps*) and Tarim Schizothoracin (*Schizothorax biddulphi*) are two recognized types of fish of Schizothoracinae which belong to the family Cyprinidae. Big-head Schizothoracin and Tarim Schizothoracin are sympatrically distributed in the Tarim River basin in Xinjiang, China, and have been listed as first-class and second-class national key protected animals, respectively. Based on morphological characteristics, *Schizothorax esocinus* (another Schizothoracin that occurs in the Tarim River basin) is speculated to be hybrid offspring of Big-head Schizothoracin and Tarim Schizothoracin, but there is no direct genetic evidence for this point. In this study, the hybridization status of *Schizothorax esocinus* was confirmed by comparing genetic constitutions of mitochondrial DNA (mtDNA) and inter transcribed spacer (ITS2) encoded by the nuclear genome. Extensive hybridization and introgression between Big-head Schizothoracin and Tarim Schizothoracin was detected. Since both Big-head Schizothoracin and Tarim Schizothoracin are critically endangered fishes, risk management due to hybridization is recommended to be considered as a part of current conservation programs.

**Abstract:**

Big-head Schizothoracin (*Aspiorhynchus laticeps*) and Tarim Schizothoracin (*Schizothorax biddulphi*) are locally sympatric in the Tarim River Basin. Although another Schizothoracin (*Schizothorax esocinus*) in Tarim River basin has been speculated to be hybrid offspring of Big-head Schizothoracin and Tarim Schizothoracin, there was no genetic evidence. Previous studies on the genetics and evolution of Schizothoracins in Xinjiang Province were mostly based on mitochondrial DNA (mtDNA), whose characteristics of maternal inheritance made it hard to answer the question of whether there was hybridization and introgression between Big-head Schizothoracin and Tarim Schizothoracin. In this study, cytochrome b (*cytb*) gene of mtDNA and internal transcribed spacer 2 (ITS2) that is encoded by the nuclear genome were genotyped within the entire samples at the same time. Our results confirmed that *Schizothorax esocinus* was the hybrid offspring of Big-head Schizothoracin and Tarim Schizothoracin. The heterozygous ITS2 genotypes and/or *Aspiorhynchus laticeps*-like mtDNA were also detected in a subset of samples that should have been identified as pure *Schizothorax biddulphi* based on morphology. The ITS2 is characterized by multi-copy, concert evolution, and biparental inheritance. Thus, by comparing with mtDNA data, broad-scale bidirectional hybridization and introgression between Big-head Schizothoracin and Tarim Schizothoracin were revealed. Although interspecific hybridization may play a positive role in ecology and evolution, interspecific hybrids could threaten their parental species by the swamping of genetics and demography. As both parents of hybridization are critically endangered fishes, in this case, it is urgently necessary to strengthen the scientific assessment of the risks of the hybrids and the control of the hybridization and introgression between *Aspiorhynchus laticeps* and *Schizothorax biddulphi* in the Tarim River Basin.

## 1. Introduction

The important role of interspecific hybridization in ecology and evolution is increasingly recognized during the past decades. Hybridization among species can provide richer genetic variability, helping organisms adapt quickly to changing environments and spread into new habitats [1]. Hybridization is also the main pathway of speciation in evolution, which can lead to the formation of new taxa and increase species diversity [2,3]. Examples of hybridization are widespread—roughly 25% of plants and 10% of animals undergo hybridization during the speciation process [1]. However, interspecific hybridization breaks down the boundaries between genetically distinct species [1,4], which can adversely affect parental species or other organisms in communities. The hazards of interspecific hybridization are particularly severe for endangered species, for it may contribute to endangered species’ decline or even accelerate them towards extinction [4,5]. Genetic extinction of rare species may occur through genetic assimilation due to introgressive hybridization from more common close relatives [6]. Another main threat to endangered species is demographic swamping, where reproductive effort is ‘wasted’ on hybridization [7]. Many cases of interspecific hybridization have been documented in fish, and members of the Cyprinidae family were found to hybrid relatively more frequently [8]. Morphological differences among parent species and hybrids were once taken as the main indicators of hybridization in fish [1,8]. The development of genetic markers, especially DNA markers, provides many powerful tools for identifying hybridization and could reveal hidden introgression and hybridization directions, thereby laying a foundation for exploring the mechanism of interspecific hybridization [9,10,11].

**Figure 1:**
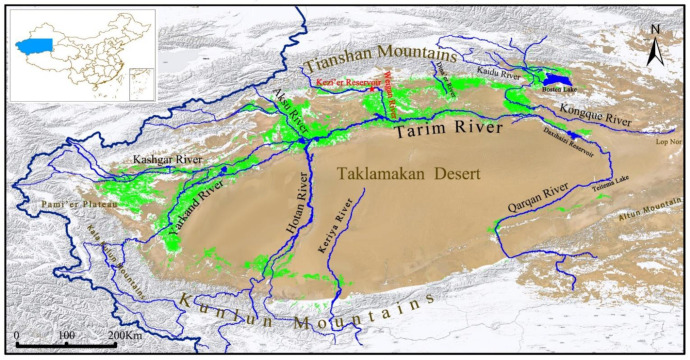
Map of Tarim River Basin. The red star indicates the sampling site of this study (Kezi’er Reservoir). The green areas in the figure indicate the main oasis within the basin. *Aspiorhynchus laticeps* and *Schizothorax biddulphi* were once widely distributed in major tributaries of Tarim River, but Weigan River (Red font) has become the last habitat of *Aspiorhynchus laticeps* now.

The fish of the subfamily Schizothoracinae (family Cyprinidae) are widespread in the high-altitude rivers and lakes in the Qinghai–Tibet Plateau and its surrounding areas [12]. Big-head Schizothoracin (*Aspiorhynchus laticeps*) and Tarim Schizothoracin (*Schizothorax biddulphi*) are sympatrically native fishes in the Tarim River basin (Figure 1) and are usually defined as flagship species in Xinjiang, China [13]. Due to multiple anthropogenic stressors, such as climate change, habitat loss, degradation, or fish translocations, the population of Big-head Schizothoracin has declined dramatically in the late twentieth century [14,15,16]. Big-head Schizothoracin has been listed as one of the four first-class protected fish species in China in 1988 and was included in the “*Red Book of Endangered Animals in China*” in 1998. Big-head Schizothoracin was once considered to be extinct, but it was rediscovered in the 1990s [10]. From then onwards, the Big-head Schizothoracin were restricted to the Kezi’er Reservoir in the Weigan River [17,18,19,20]. Tarim Schizothoracin was also included in the “*Red Book of Endangered Animals of China*” in 1998 and was listed as a second-class protected species of Xinjiang Autonomous Region in 2004 and upgraded as a second-class national protected wild animal in 2021. This fish species previously widely inhabiting the mainstream and tributaries of the Tarim River can be only found in some tributaries (i.e., the Aksu River, Weigan River, Yarkand River, and Hotan River, see Figure 1) of the Tarim River [17,18]. In arid or semi-arid regions, habitat shrinkage due to land use and climate change may reduce the survival of the endangered species [19].

There have been a certain number of studies on the conservation genetics of Schizothoracians in the Tarim River Basin [20,21,22,23,24,25,26]. However, almost all of the previous studies were based on mitochondrial DNA (mtDNA) markers alone. Animal mtDNA has a number of advantages in terms of rapid evolution, lack of recombination, and ease for PCR amplification, which made it widely used in conservation and evolutionary genetics. mtDNA is typically maternal inherited [27]. Though it can help to identify the female parent of hybridization, there is an obvious deficiency in accurately assessing the genetic contributions of both parents in hybridization and introgression [28]. Nuclear ribosomal RNA genes of cyprinids are similar to those of most eukaryotes, which are encoded by nuclear ribosomal DNA (nrDNA) as polygenic families of tandem repeats [29]. Each 45s rDNA repeat unit contains genes for 18S rDNA, 5.8S rDNA, and 28S rDNA, and internal transcribed spacers (ITS) between the coding genes. The coding regions of ribosomal RNA genes are highly conserved. Even the sequences of distant species are usually completely consistent, which facilitates the design of conservative PCR primers. Sequences of the internal transcribed spacer evolve relatively quickly as non-coding regions and can provide more mutation sites between species [29]. Because the repeat unit of nrDNA undergoes a concerted evolution manner, this suggests that there is extremely little sequence variation of the internal transcribed spacers within the same species but a high frequency of sequence variation between different species [30]. Internal transcribed spacer 2 (ITS2) is very useful for species identification, which is located between 5.8S rDNA and 28S rDNA [31,32]. Due to ITS2 and mtDNA holding different genetic and evolutionary properties, comparison of them in the same samples has been one of the classic methods for elucidating interspecific hybridization [33,34].

**Figure 2:**
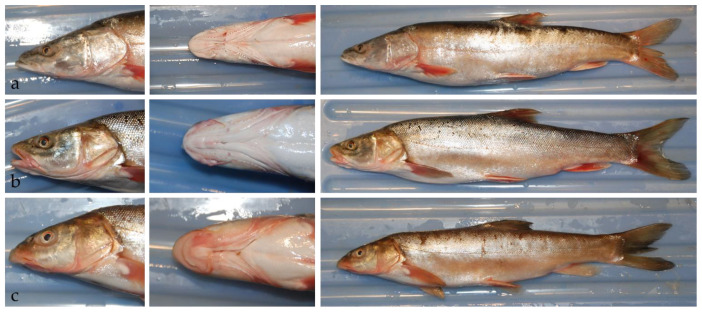
Morphological differences among *Aspiorhynchus laticeps* (**a**)*,* *Schizothorax esocinus* (**b**) and *Schizothorax biddulphi* (**c**). The head of *A. laticeps* is larger, with a flattened snout (known as Flat-snouted fish) and only 1 pair of barbels. The mouth of *A. laticeps* is at the front, and the lower jaw is longer than the upper jaw. The head of *S. biddulphi* is smaller, with a spiky snout (known as Spiky-billed fish) and 2 pairs of barbels. The mouth of *S. biddulphi* is lower, and the upper jaw is longer than the lower jaw. The morphological characteristics *S. esocinus* (known as Flat-billed fish) fall somewhere intermediate *A. laticeps* and *S. biddulphi*. The mouth of *S. esocinus* is at the front with 2 pairs of barbels, meanwhile, the upper and lower jaws are nearly equal in length.

There is another Schizothoracin (*Schizothorax esocinus*) inhabiting the Tarim River Basin, whose morphological characteristics are intermediate between Big-head Schizothoracin and Tarim Schizothoracin (Figure 2) [13]. “*Fishes of Xinjiang*” inferred that *Schizothorax esocinus* may be hybrid offspring between Big-head Schizothoracin and Tarim Schizothoracin [9]. Current conservation policies and actions for Schizothoracins in the Tarim River Basin have not considered the existence of interspecific hybridization between them. If *Schizothorax esocinus* is the interspecific hybrids of Big-head Schizothoracin and Tarim Schizothoracin, conservation programs of Schizothoracins in Tarim River basin might need to be redesigned. Based on the comparative analysis of cytochrome b (*cytb*) gene of mtDNA and ITS2 region of the nuclear genome, we provided evidence for broadscale hybridization and introgression between Big-head Schizothoracin and Tarim Schizothoracin in Tarim River Basin and confirmed the hybrid status of *Schizothorax esocinus*.

## 2. Materials and Methods

### 2.1. Sample Collection

In 2009, fin clip specimens (*n* = 69) of Schizothoracins were collected from the Kezi’er Reservoir located at the upper reaches of the Weigan River (a tributary of the Tarim River). We have recognized the differences between “*Fauna Sinica*” and “*Fishes of Xinjiang*” on species delimitation of genus *Schizothorax* in Tarim River Basin. The former said that Tarim Schizothoracin is the only valid species of *Schizothorax* in Tarim River Basin, but five species of genus *Schizothorax* were recorded in the latter [12,13]. In order to avoid missing out on important morphological characters, we followed the classification standard of “*Fishes of Xinjiang*” during sample collection [13]. The numbers of each taxon were as follows: 5 *Aspiorhynchus laticeps*, 4 *Schizothorax esocinus*, 8 *Schizothorax irregularis*, 32 *Schizothorax eurystomus*, 19 *Schizothorax biddulphi,* and 1 *Schizothorax barbatus*. However, results of this study (see below) supported the viewpoint of “*Fauna Sinica*” [12], except for confirming that *Schizothorax esocinus* was hybrid offspring of *Aspiorhynchus laticeps* and *Schizothorax biddulphi* [13]. Thus, 8 *Schizothorax irregularis*, 32 *Schizothorax eurystomus*, 19 *Schizothorax biddulphi* and 1 *Schizothorax barbatus* were combined into *Schizothorax biddulphi* (total of 60 individuals). Total DNA was extracted from the alcohol-preserved fin tissues by phenol–chloroform extraction method.

### 2.2. Amplification and Analysis of cytb Gene

**Figure 3:**
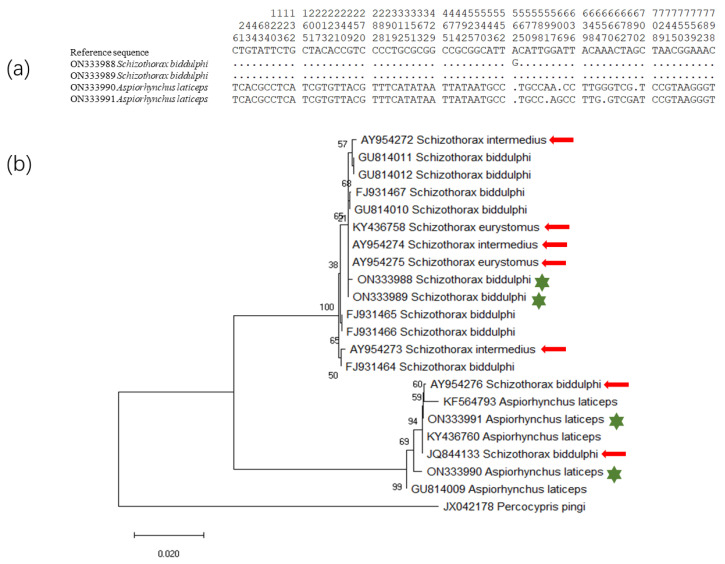
Comparative analysis of *cytb* gene of *Aspiorhynchus laticeps* and *Schizothorax biddulphi*. (**a**) Variable sites of aligned four haplotypes. (**b**) NJ phylogenetic tree of *cytb* haplotypes in Schizothoracin with *cytb* sequence of Jinsha perch carp (*Percocypris pingi*) as outgroup. Red arrows marked the wrongly clustered sequence, while green asterisk represented four haplotypes of this study.

Cytochrome b (*cytb*) gene was amplified with primer pair L14724 and H15915 reported by Xiao et al. [35] with slight modification. Five internal primers were designed to verify and sequence PCR products. The details of primers are listed in Table 1. PCR reaction mixture was 32 μL, including 1× PCR mixture (Cowin Bioscience, Cambridge, MA, USA), 10 pmol each primer and about 50 ng genomic DNA. Amplifications were performed on the GeneAmp PCR system 9700 (Applied Biosystems, Waltham, MA, USA) with following program: pre-denaturation at 94 °C for 2 min, followed by 35 cycles of denaturation at 94 °C for 30 s, renaturation at 58 °C for 30 s, extension at 72 °C for 1 min, a final hold at 72 °C for 7 min. After inspection by 1% agarose gel electrophoresis, PCR products were submitted for sequencing on 3730 XL sequencer at Genewiz company. All sequences were aligned by using cluster X software [36] and trimmed to the same length. Dnasp v6 software [37] was used to calculate the number of haplotypes, haplotype diversity, nucleotide diversity, number of polymorphic sites and other parameters. Several other related sequences were retrieved from Genbank and included in phylogenetic analysis (Figure 3). With *cytb* sequence of Jinsha perch carp (*Percocypris pingi*) as outgroup, a phylogenetic tree was constructed using the Neighbor-joining (NJ) method in mega-X [38] software. The neighbor-joining trees are based on the maximum composite likelihood model. Branch supports for NJ trees tree were measured by bootstrap analysis with 1000 random replicates.

### 2.3. Amplification and Analysis of ITS2 Region

Internal transcribed spacer 2 (ITS2) of each sample was amplified using primer pair of 5.8S-F and 28S-R, which were designed based on sequences of goldfish (*Carassius auratus*) and common carp (*Cyprinus carpio*), which were also used in sequencing reactions. Then, according to the sequencing results, an internal primer (ITS2_239R) was designed to detect the length polymorphic of ITS2 length by capillary electrophoresis. The information on primers was also listed in Table 1. Except for using different primers, PCR reaction mixture of ITS2 gene is consistent with that of *Cytb* gene. The reaction procedure was pre-denaturation at 98 °C for 30 s, 35 cycles of denaturation at 98 °C for 10 s, annealing at 58 °C for 30 s, extension at 72 °C for 30 s, and a final extension at 72 °C for 7 min. The amplified products were inspected by 1% agarose gel electrophoresis, and positive PCR products were sent to Genewiz Company for sequencing.

Sequencing results were viewed by FinchTV (Geospiza Inc., Seattle, WA, USA), then successful sequences were aligned by Clustalx [36] software to identify haplotypes and polymorphic sites. According to the alignment, a primer with pigtail [39] was designed in ITS2 (Table 1) to pair with Rox fluorescent-labeled 5.8S-F under the same PCR conditions. After amplified products were added Liz500 as internal standard, the fragment length was detected by ABI platform. The length of fragments was calculated by Genemarker 2.4.2 (Softgenetics, State College, PA, USA) software, and the ITS2 genotype of each individual was recorded.

## 3. Results

### 3.1. Sequence Variation of cytb Gene

Partial sequences (798 bp) of *cytb* gene were successfully sequenced for all specimens, and a total of four haplotypes were obtained from 69 individuals (GenBank ON333988-ON333991). These four haplotypes were separated into two clades (Figure 3), one clade for *A. laticeps* and the other for *S. biddulphi*. All parameters of genetic diversity were listed in Table 2. Two haplotypes of *A. laticeps*-type mtDNA were found in 15 samples with six variable sites among them, other two haplotypes of *S. biddulphi*-type mtDNA were found in the remaining 54 samples with only one variable site. When the four haplotypes were compared together, the number of variable sites increased to seventy, indicating the deep divergence between Big-head Schizothoracin and Tarim Schizothoracin. The distribution of mitochondrial haplotypes for each species was summarized in Table 3.

### 3.2. Sequence Features of ITS2 Region

**Figure 4:**
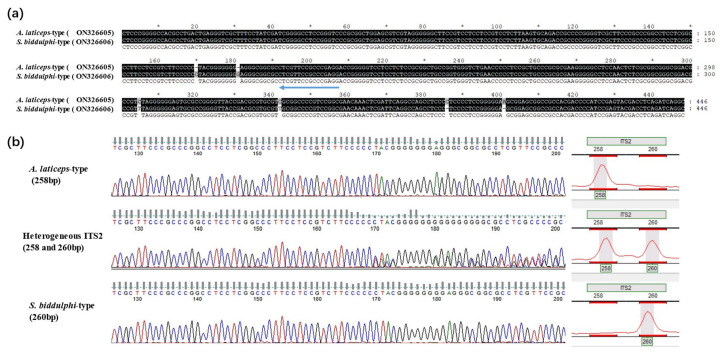
Genotypes of internal transcribed spacer 2 (ITS2) in *Aspiorhynchus laticeps* and *Schizothorax biddulphi.* (**a**) Differences in ITS2 between *Aspiorhynchus laticeps* and *Schizothorax biddulphi* and position of inner primer (indicated by the arrow) were displayed. (**b**) Overlapping peak causing two different lengths of ITS2 sequences in the same individual result in sequencing failure.

PCR products of ITS2 were directly sequenced, and a total of 446 bp of the sequence was obtained after alignment and trimming (GenBank ON326605-ON326606), including 33 bp of 3’ end of 5.8S rDNA, 26 bp of 5’ends of 28S rDNA, and 387 bp of complete ITS2 region. A total of six variable sites were detected (Figure 4), including four indels and two substitutions. All variable sites are located within the ITS2 region, rather than in flanking 5.8S rDNA or 28S rDNA. Among these sequences, there were only two haplotypes, one for Big-head Schizothoracin and the other for Tarim Schizothoracin. After alignment, it was not difficult to find that both lengths of the ITS2 sequence were 387 bp. Before about 180 bp, ITS2 of Big-head Schizothoracin had 2 more bp than that of Tarim Schizothoracin, but the numbers just flip over after that. In some individuals, overlapping peaks appeared at about 170 bp and caused sequencing to fail. We speculated that the failure of sequencing may reflect two different lengths of ITS2 sequences in these individuals. An internal primer (Table 1) was designed to detect the length variation of the ITS2 region. As we envisioned, two fragments of 258 bp and 260 bp were detected (Figure 4). There were only 258 bp fragments in Big-head Schizothoracin, while 260 bp fragments were dominant in Tarim Schizothoracin. Additionally, all four *Schizothorax esocinus* and a part of Tarim Schizothoracin showed both 258 bp and 260 bp fragments.

### 3.3. Comparative Analysis of Morphological, Mitochondrial and Nuclear Data

This study found only two clades of mtDNA, one for Big-head Schizothoracin and the other for Tarim Schizothoracin, which did not support the view of “Fishes of Xingjiang” on species delimitation of *Schizothorax* in Tarim River basin [13]. The results from ITS2 genotypes were consistent with these of mtDNA. In all samples that were identified as Big-head Schizothoracin based on morphology, both mtDNA and ITS2 belong to Big-head Schizothoracin types. All four *Schizothorax esocinus* samples harbored heterozygous ITS2 genotypes. Meanwhile, three out of the four samples had mtDNA of Big-head Schizothoracin type and the rest had mtDNA of Tarim Schizothoracin type. It was found that introgression of Big-head Schizothoracin type mtDNA into an individual of *Schizothorax biddulphi* with homozygous ITS2 genotype of Tarim Schizothoracin. Among the remaining 59 Tarim Schizothoracin, 48 samples have homozygous ITS2 genotype of Tarim Schizothoracin type. Furthermore, all mtDNA of these 48 samples were Tarim Schizothoracin type. Mitochondrial DNA of Big-head Schizothoracin type and Tarim Schizothoracin type each have accounted for about half of the remaining 11 (6 and 5, respectively) samples whose ITS2 was heterozygous. We also noted that published mtDNA sequences from Tarim Schizothoracin and Big-head Schizothoracin were present in both clades wrongly, indicating that morphological identification results were not always consistent with mtDNA and ITS2 genotypes (Figure 3).

## 4. Discussion

In this study, only two types of mtDNA were found—one for Big-head Schizothoracin and the other for Tarim Schizothoracin. A similar situation has been also observed in ITS2 regions. According to the “*Fishes of Xinjiang*”, there were five species of the genus *Schizothorax* in the Tarim River Basin: *Schizothorax biddulphi, Schizothorax esocinus*, *Schizothorax irregularis*, *Schizothorax eurystomus* and *Schizothorax barbatus* [13]. However, our results showed that both mtDNA and ITS2 sequences of *Schizothorax* species in Tarim River Basin only clustered into one clade, which indicated that there was no significant genetic differentiation among them. As mentioned above, “*Fauna Sinca*” recognized Tarim Schizothoracin as the only valid species of *Schizothorax* in Tarim River basin [12]. A recent study on the genetic structure of 419 *Schizothorax* fishes based on the COI gene revealed that the genetic distance between *Schizothorax* samples from the main tributaries of the Tarim River is less than 0.005, and no significant “barcode gap” is formed [40]. Both mtDNA data of that study and our work supported the view of “*Fauna Sinca*” that there is only one valid species of genus *Schizothorax*- Tarim Schizothoracin in this drainage. The species identification of the *Schizothorax* genus in the “*Fishes of Xinjiang*” mainly focused on some minor differences in morphological characteristics of mouths and lips [13], which may not reflect the real boundaries of the species. Our study reveals that the ITS2 region of the nuclear genome also supports this point.

Genotypes of mtDNA and ITS2 in five samples of *Aspiorhynchus laticeps* herein are all Big-head Schizothoracin types. Heterozygous ITS2 genotype and two types of mtDNA were found in the four samples identified as *Schizothorax esocinus* based on morphology, which suggested that they were hybrids of Big-head Schizothoracin and Tarim Schizothoracin. The situation is more complicated in the samples of morphological Tarim Schizothoracin. In those samples with homozygous Tarim Schizothoracin ITS2 genotype (49/60), most mtDNA were Tarim Schizothoracin type (48/49) and only one was Big-head Schizothoracin Type (1/49). Among ITS2 heterogeneous individuals, Tarim Schizothoracin type mtDNA (5/11) and Big-head Schizothoracin type mtDNA (6/11) each account for about half. Since mtDNA is maternally inherited [27] and ITS2 encoded by the nuclear genome with bi-parental inheritance [29], the results indicated that both Big-head Schizothoracin and Tarim Schizothoracin could be the female parent of a hybrid and/or hybrid can backcross with both parental species. No later than the 1970s, *Schizothorax esocinus* has been listed in “*Fishes of Xinjiang,*” which was speculated to be the product of interspecies hybridization between Big-head Schizothoracin and Tarim Schizothoracin [13]. The environmental changes and human activities have changed the ecology and environment of Tarim Basin dramatically over the past 50 years [41]. There was no large-scale domestication, artificial breeding and stock enhancement of indigenous Schizothoracins in the Tarim River Basin before the 1990s, so we tend to agree that there may be natural interspecific hybridization between Big-head Schizothoracin and Tarim Schizothoracin. However, environmental changes and human intervention may increase the risk of hybridization and introgression.

Unexpectedly, a higher proportion of individuals (11/60) with heterozygous ITS2 genotype was also found in the samples that were morphologically identified as Tarim Schizothoracin. We thought that *Schizothorax esocinus* might be the first generation of hybridization between Big-head Schizothoracin and Tarim Schizothoracin or an early generation of backcrossing, leading to *Schizothorax esocinus* morphological similarity to both parents [13]. However, these Tarim Schizothoracin-like individuals are likely to be the descendants of gradual introgression. It should be noted that the ITS2 genotype of one Tarim Schizothoracin was also a homozygous Tarim type, whereas the mtDNA is Big-head Schizothoracin type, which means introgression of mtDNA from Big-head Schizothoracin into Tarim Schizothoracin. We also found evidence for mtDNA introgression from the Big-head Schizothoracin into Tarim Schizothoracin from publicly available data. The only complete sequence of Tarim Schizothoracin mitochondrial genome that can be retrieved from GenBank is highly similar to that of Big-head Schizothoracin, rather than to partial sequence and mitogenome of “real” Tarim Schizothoracin (Figure 3). Using mitochondrial control region (CR) and cytochrome oxidase subunit 1 (*COI*) gene to analyze the genetic diversity and population differentiation of Tarim Schizothoracin, previous studies found that there was a deep divergence between the population of Qarqan River and the other populations of Tarim Schizothoracin [26,40]. These phenomena reflected introgression of the Big-head Schizothoracin’s mtDNA into a certain number of morphologically identified Tarim Schizothoracin. All above evidence suggested extensive hybridization and introgression between Tarim Schizothoracin and Big-head Schizothoracin has occurred in the Tarim River Basin.

We thought that interspecific hybridization of Big-head Schizothoracin and Tarim Schizothoracin is likely to occur naturally, but the potential harms of interspecific hybridization should be brought to the attention of the public for several reasons. First, populations of Big-head Schizothoracin and Tarim Schizothoracin have shrunk to extreme atrophy and are difficult to find in many of their original habitats [15,16,17,18], and interspecific hybridization is likely to jeopardize the survival of both parental species. Second, the ecological environment of the Tarim River Basin has undergone drastic changes in the past and is still evolving now [41]. The complex relationships between the great changes in the ecological environment and the risk of hybridization and introgression remain unclear. Therefore, the risk is still difficult to evaluate. Third, both indigenous Schizothoracins have undergone large-scale stock enhancement [42], and the presence of extensive interspecies hybridization and introgression indicated that the parents used in the breeding and stocking program are likely to be the descendants of hybridization and introgression. If the hybrids were mistakenly used as breeding parents during artificial proliferation and stocking, it will undoubtedly accelerate the mixture of the two gene pools and even threaten the survival of the two parental species. Due to the fact that misjudgment hahas been found in several studies [26,40,43], we thought it very likely to happen. In a word, it is necessary to monitor the risk of interspecific hybridization of two endangered Schizothoracins to avoid broad-scale interspecific hybridization with irreparable consequences.

## 5. Conclusions

For the first time, this study provides molecular evidence for the hybrid status of *Schizothorax esocinus,* which is the morphologically suspicious hybrid of Big-head Schizothoracin and Tarim Schizothoracin. Our study also showed that hybridization and introgression between the two Schizothoracins were much broader than the previous speculation, and even some individuals were morphologically difficult to distinguish from their parent, Tarim Schizothoracin. We suggested scientifically assessing and controlling the risks of hybridization and introgression in conservation programs of Schizothoracins in the Tarim river basin.

## Figures and Tables

**Table 1:** PCR and genotyping primers used in this study.

Primers	Sequence (5′-3′)	Reference
L14724	GACTTGAAGAACCACCGTTG	[35]
H15915	CTCCGATCTCCGGATTACAAGAC	[35]
15353F	CGCTAACGAYGCACTAGTTGA	This study
ALCB_104F	GCCTTCTGGGATTATGCTTAGC	This study
ALCB_1005R	AGGATAACTATGTCTGCAACC	This study
ALCB_581R	CGGTTGCGTCGGCAATGACA	This study
ALCB_494F	GAGGCGGATTCTCGGTAGAT	This study
5.8S_F	CAGGACACATTGATCATCGACAC	This study
28S_R	CCGCTACTGAGGGAATCCTTGTT	This study
ITS2_239R	GTTTCCTCGAGCGGAACGAG	This study

**Table 2:** Diversity parameters of *cytb* gene in two Shcizothoracins from Tarim River Basin.

	*n*	*h*	Hd	π (%)	K	S
*Aspiorhynchus laticeps*-type	15	2	0.343 ± 0.128	0.172 ± 0.064	1.371	4
*Schizothorax biddulphi*-type	45	10	0.283 ± 0.068	0.035 ± 0.009	0.283	1
Total	69	4	0.533 ± 0.059	2.935 ± 0.477	23.425	70

Note: *n*: Number of individuals; *h*: Number of Haplotypes; Hd: Haplotype diversity; π: Nucleotide diversity; K: Average number of nucleotide differences; S: Number of segregating sites.

**Table 3:** Taxon distribution of mitochondrial haplotypes for Shcizothoracins from Tarim River Basin.

Taxon	ITS2 Genotype	*A. laticeps* Type (ON333990)	*A. laticeps* Type (ON333991)	*S. biddulphi* Type (ON333988)	*S. biddulphi* Type (ON333989)	Total
*A. laticeps* ( homogeneous ITS2)	258 bp	2	3			5
*S. esocinus* (heterogeneous ITS2)	258 bp and 260 bp		3		1	4
*S. biddulphi* (heterogeneous ITS2)	258 bp and 260 bp	1	5	1	4	11
*S. biddulphi* (homogeneous ITS2)	260 bp		1	8	40	49
Total		3	12	9	45	69

## Data Availability

All sequences have been deposited to GenBank (accession numbers ON333988-ON333991 and 326605-ON326606).
